# Metal Oxide Nanoparticles Against Bacterial Biofilms: Perspectives and Limitations

**DOI:** 10.3390/microorganisms8101545

**Published:** 2020-10-07

**Authors:** Liubov Shkodenko, Ilia Kassirov, Elena Koshel

**Affiliations:** 1 Microbiology Lab of SCAMT Institute, ITMO University, Lomonosova st. 9, 191002 St. Petersburg, Russia; shkodenko@scamt-itmo.ru (L.S.); kassirovilya@gmail.com (I.K.); 2 Department of Epidemiology, Pasteur Institute, 197101 St. Petersburg, Russia

**Keywords:** metal oxide nanoparticles, nanocomposites, biofilm, antibiofilm properties, antibacterial properties, mutagenicity

## Abstract

At present, there is an urgent need in medicine and industry to develop new approaches to eliminate bacterial biofilms. Considering the low efficiency of classical approaches to biofilm eradication and the growing problem of antibiotic resistance, the introduction of nanomaterials may be a promising solution. Outstanding antimicrobial properties have been demonstrated by nanoparticles (NPs) of metal oxides and their nanocomposites. The review presents a comparative analysis of antibiofilm properties of various metal oxide NPs (primarily, CuO, Fe_3_O_4_, TiO_2_, ZnO, MgO, and Al_2_O_3_ NPs) and nanocomposites, as well as mechanisms of their effect on plankton bacteria cells and biofilms. The potential mutagenicity of metal oxide NPs and safety problems of their wide application are also discussed.

## 1. Introduction

Biofilm is a wide-range life form of bacteria that consists of the association of microorganisms cultures and extracellular polymer matrix (EPM), a complex biochemical mixture of polysaccharides, proteins, glycopeptides, nucleic acids, and lipids [1,2]. This slimy three-dimensional biopolymer is heterogeneous in different layers and contains structures similar to transport and water channels [3]. In biofilm formation, there are three main stages: adhesion, colonization, and maturation [4,5]. From the mature biofilm, the plankton microorganisms are isolated or dispersed into the environment. Thus, biofilm is a complex three-dimensional biological structure with a higher microbial life organization, in many respects, similar to a multicellular organism [6].

The primary function of biofilm is to protect the microorganism inside it from unfavorable physical, chemical, and biological factors in the environment, such as temperature, drying, ultraviolet radiation, biocides, humoral, and cellular factors of immunity [4]. Therefore, biofilms are stable, stress-resistant structures, which are difficult to destroy. They can cause many problems in various areas: water treatment (biofilm can disrupt the microbial community of active silt) [7]; food production (biofilm formation by pathogenic microorganisms and spoilage microorganisms) [8]; implantology (biofilms are capable of infecting medical devices: intravenous catheters, vascular prostheses, heart valve prostheses, urinary catheters, joint prostheses, pacemakers, and contact lenses) [9]; treatment of chronic diseases (according to the Center for Disease Control, biofilms cause 65% of chronic infections, and the National Institute of Health increases this index to 80%) [10,11]. All these problems have emerged since standard antibacterial therapy is practically ineffective against biofilms.

In most cases, the concentrations of antibiotics needed to kill the floating (planktonic) forms of bacteria can be 1000 times less than the required concentration for the complete removal of biofilms [10]. This tolerance to antibiotics causes the transition of infections caused by biofilms to a chronic form [7,12,13]. Initially, it was suggested that a unique biofilm structure could be a physical barrier to the inward penetration of antibiotics. However, more recent studies have shown that the diffusion of antibiotics is not hampered by EPM [14,15]. A further hypothesis is that after binding to polysaccharides, proteins, and DNA present in the biofilm, antibiotics may no longer be biologically active or cannot reach a necessary concentration for effective bacterial destruction [16].

Additionally, there is a gradient of oxygen concentration and nutrients inside the biofilm, which makes antibiotics, whose action is associated with metabolic disorders, much less effective against bacteria due to slowing or stopping metabolism [17]. Besides the high biofilm resistance to antibiotics, there has been an increased amount of information about biofilms formed by multidrug-resistant strains over the past decade. The latter further enhances the problem of combating biofilms in medicine and other areas.

The problems described above cause an urgent need to develop new ways of inhibiting biofilm growth and elimination. One of the potentially successful strategies may be transitioning from standard therapy to high-tech solutions based on nanomaterials.

Nanoparticles (NPs) are considered a promising tool for the treatment of bacterial biofilms. It is due to antibiotic resistance mechanisms not being effective against NPs [18]. Among many NPs, the most promised and widely studied are metal oxide nanoparticles, such as TiO_2_, Fe_3_O_4_, ZnO, CuO [19], and some mixed metal oxides [20,21,22]. It has been shown that many metal oxide NPs exhibit biological properties much better than the NPs of the parent metals. That is why the metal oxide NPs provoked the highest interest in the scientific community [23,24].

In this review, we collected and systematized all of the information received in recent years on the effectiveness of using metal oxide NPs against biofilms. A comparative analysis of CuO, Fe_3_O_4_, TiO_2_, ZnO, MgO, and Al_2_O_3_ NPs and nanocomposites of oxides was conducted. Potential mutagenicity and biosafety problems of these nanomaterials are also discussed.

## 2. The Mechanism of NPs Interaction with Biofilms

EPM of biofilms is heterogeneous from the point of physicochemical properties of a structure containing many polymer molecules carrying a charge [25]. Therefore, biofilm can be considered a three-dimensional filter capable of capturing organic molecules, ions, and NPs.

**Figure 1:**
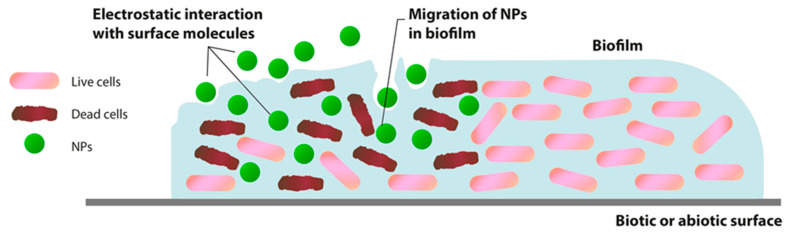
Interaction between metal oxides nanoparticles (NPs) and biofilm.

The interaction between NPs and biofilm can be considered as a three-stage process: (1) transfer of NPs in the vicinity of the biofilm; (2) attachment to the biofilm surface; and (3) migration in biofilms (Figure 1). The implementation of each stage is conducted by many factors: the physicochemical characteristics of NPs, EPM, and the environment.

Once the NPs reach the biofilm boundary, the physicochemical characteristics of the EPM determine the initial attachment of NPs to the biofilm surface and their subsequent movement in the matrix. The initial addition of NPs to the outermost surface of biofilms can be affected by various physicochemical interactions.

Primarily, the interaction between NPs and biofilm is determined by their electrostatic characteristics. These features depend on the zeta potential of NPs and the charge of the biofilm matrix [26,27,28,29]. The majority of bacteria have a polyionic biofilm matrix due to the presence of uronic acid or metal-bound pyruvate with the functions of carboxylic acid and residual phosphate or rarely sulfate [30,31]. This negatively charged matrix can interact with positively charged metal ions and organic compounds through electrostatic forces [32,33].

Successfully associated NPs with EPM on the biofilm surface can penetrate deep into the biofilm at different rates. The penetration of NPs and movement within the biofilm is considered to be primarily due to diffusion [34]. In this case, the NPs’ diffusion inside the biofilm may depend on the size of its pores [28], water channels presence [35], the charge of NPs and EPM [34], hydrophobicity of the environment [36], the chemical gradient within the matrix. The EPM pore spaces containing water can have different ion concentrations. Ions and organic molecules diffuse and penetrate the biofilm, move, and are distributed through these pore spaces. This gives a plausible possibility that the interval between EPM pores will be especially crucial in this process. However, this variability on the scale of nanometers is not sufficiently characterized and understandable [37].

Thus, the penetration and migration of NPs inside the biofilm will be determined mainly by the charge and size of the particles and the composition and structure of EPM. However, many details of this interaction have yet to be determined.

## 3. Effects of Metal Oxide NPs on Plankton Cells and Biofilm

**Figure 2:**
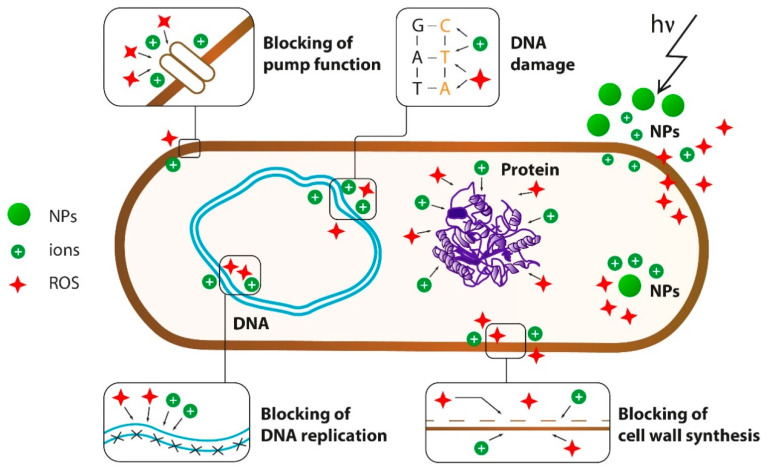
Effects of metal oxide NPs on the bacterial cell. Brown line—cell surface (cell wall and cell membrane), blue line—DNA, arrow—electromagnetic irradiation.

Three main mechanisms of antibacterial effects of NPs are well known: (1) mechanical damage to the cell wall as a result of electrostatic interaction, (2) oxidative stress as a result of the generation of reactive oxygen species (ROS), and (3) disruption of proteins functions and cell structures as a result of metal cations release [18] (Figure 2).

**Figure 3:**
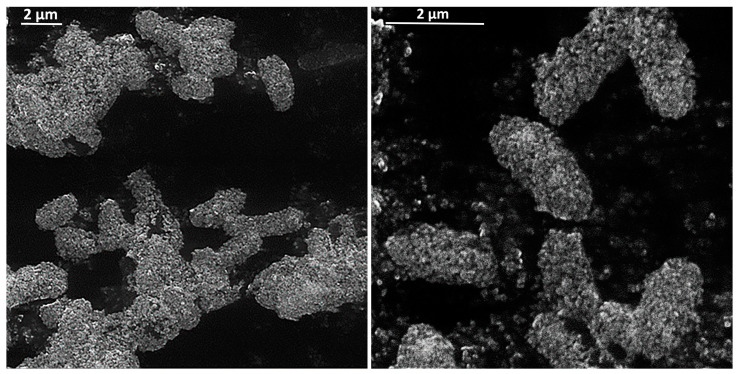
Electrostatic and Van der Waals interactions between magnetite (Fe_3_O_4_) NPs and bacterial cells: NPs adhere to the cell wall and form a magnetite shell. The images obtained by scanning electron microscopy (Tescan VEGA 3, Brno, Czech Republic).

For negatively charged bacteria, adhesion on metal oxide NPs increases due to a positively charged surface of NPs [38]. Figure 3 shows how actively NPs interact with the cell wall on the example of magnetite. In this way, metal oxide NPs binds to the cell wall through electrostatic and Van der Waals interactions, in particular, to cell membrane proteins that disrupt bacteria functions. MgO NPs also show strong interactions not only with the cell surface but also with spores [39,40].

The primary mechanism of bacterial cell destruction under the NPs’ influence is currently considered to be the induction of oxidative stress through the generation of ROS under electromagnetic irradiation of NPs [41]. The generation of ROS induces oxidative stress in the cell, as a result of which it dies. In most cases, ROS production is also directly related to the release of cations. In particular, Fe_3_O_4_ NPs release Fe^2+^ ions, which cause the generation of ROS after a reaction with hydrogen peroxide (Fenton reaction) [42]. Copper ions can also disrupt biochemical processes and significantly damage nucleic acids [43]. It is assumed that after the specific binding of copper to DNA, repeated cyclic redox reactions generate several OH^−^ radicals near the binding site, causing multiple damages to the nucleic acids, but in some microorganisms, copper oxidative damage to the genetic material may occur through the Fenton mechanism [44]

Calcium and magnesium oxides can generate superoxide radical O2, whereas zinc oxide NPs produce H_2_O_2_ and hydroxyl radical OH^−^ under ultraviolet and visible light, but not O_2_ [45,46]. The molecules of OH^−^ and O_2_ cannot penetrate the cell membrane due to their charges or reactivity [47], and probably remain on the cell surface, whereas H_2_O_2_ can penetrate bacterial cells, thereby inducing cell death [48,49]. Meanwhile, copper oxide NPs can produce all four types of reactive oxygen. Thus, CuO NPs are toxic enough for bacteria and have shown significant effects against biofilms.

TiO_2_ NPs under irradiation can generate electron-hole pairs with photoexcitation, initiate cascade oxidation–reduction reactions on the surface of TiO_2,_ and, consequently, produce ROS for further reactions [50,51]. Thus, TiO_2_ NPs inhibit bacterial growth by lipid peroxidation in the membranes, DNA damage, nucleotide and amino acid oxidation, or destruction of protein-catalytic sites by photocatalysis [52].

Despite the above, there is evidence that ROS generation does not always directly cause cell death. In particular, the analysis of gene expression has shown that ZnO NPs inhibits the expression of oxidative stress genes, despite the ROS generation. The antibacterial effect may be due to biomimetic action and other mechanisms [53].

The release of metal cations can also have a devastating effect on cells, regardless of ROS products. Different bacterial species have varying sensitivities to metal ions. Cations can interact with sulfhydryl groups in enzymes, with amine and carboxyl groups on microbial cells [54,55,56]. In particular, Zn^2+^ ions influence peptides by changing their conformation [57]. Cations can cause the mismetallation of proteins in a cell. In particular, copper ions can mismetallate proteins that require a molybdenum or iron cofactor because of their affinity for thiol ligands [58,59]. This process may affect cell metabolism due to improper assembly of biosynthetic enzymes [60]. All this leads to disruption in the functioning of cellular components and cell death in the end.

The NPs discussed in this review exhibit antibacterial properties to varying degrees by implementing the mechanisms mentioned above. Conditionally, NPs can be arranged in the following order of their decreasing antibacterial and antibiofilm properties: CuO–ZnO–MgO–TiO_2_–Fe_3_O_4_–Al_2_O_3_. Physical and antibiofilm properties of some NPs oxides are presented in Table 1.

### 3.1. CuO

The minimum inhibitory concentration (MIC) for CuO NPs against a wide range of Gram-negative and Gram-positive bacteria is 15 µg/mL to 100 µg/mL on average. [63,64,98]. In particular, MIC of CuO NPs for *E. coli* is 22 µg/mL, and for *S. aureus* is 15 µg/mL [63,64]^.^

CuO NPs inhibit the formation of biofilms and promote the eradication of already formed biofilms. It is mainly due to the toxicity of copper ions for plankton and biofilm cells. For example, CuO NPs efficiently reduced the biofilm formation by MRSA and *E. coli*. Almost all MRSA and *E. coli* biofilm cells died within four days of exposure to CuO NPs [99]. CuO NPs at a 50 µg/mL concentration significantly inhibited the growth of total oral bacteria, extracellular polysaccharide (EPS) production, and biofilm formation on the glass, acrylic dentures, and cultured human epithelial cells as models [100]. It has also been shown that CuO NPs’ inhibitory concentration on *Ralstonia solanacearum* biofilms is 125 and 250 µg/mL after an incubation period of 24 and 72 h [101]. Thus, it is evident that CuO NPs can be potentially effective against biofilms, as shown in different groups of microorganisms.

### 3.2. ZnO

For ZnO NPs, the effective antibacterial concentration increases to an average of 20–500 µg/mL [102,103,104,105,106,107,108]. For example, there was 50 µg/mL against *E. coli* [105]. However, the antibacterial properties of ZnO NPs can be enhanced by additional physical exposure. In particular, cell penetration by NPs can be amplified by ultrasound. The combination of ZnO NPs with ultrasound enhances the antibacterial effect on *S. aureus* by 76% due to generating more hydrogen peroxide [109]. Besides, ultrasonic treatment can physically promote the dissociation of cell membranes, thereby increasing the penetration of ZnO NPs into cells [18].

ZnO NPs can be considered a potential agent for the inhibition of microbial biofilms. The results of the inhibition of *S. pneumoniae* biofilm showed that the sub-MIC doses (3, 6, and 12 µg/mL) of ZnO NPs exhibited significant antibiofilm activity. The main mechanism preventing the formation of *S. pneumonia* biofilms in the presence of zinc oxide NPs is that it reduces the adhesion of cells to the surface [11]. Studies of biofilms on dentures revealed the ZnO NPs’ effectiveness in controlling the formation of biofilms *Rothia dentocariosa* and *R. mucilaginosa.* Zinc ion generation inhibits the enzymatic activity of the DapE protein involved in the synthesis of peptidoglycans, which leads to the failure of biofilm formation in the initial stage [79]. The recent studies on the effect on biofilms formed by uropathogenic strains of *E. coli* have shown that MIC concentration of NPs fully inhibited biofilm formation in 20% of isolates, and 30% of isolates reduce the optical density of biofilm formation from a moderate to weak level [80]. In this way, ZnO NPs also have excellent potential for antibacterial materials’ development.

### 3.3. MgO

MgO NPs show activity against Gram-positive and Gram-negative bacteria, spores, and viruses [110,111,112,113] at sufficiently high particle concentrations (on average 100–1200 µg/mL). In particular, MIC of MgO NPs for *E. coli* were determined to be 1 mg/mL [114].

Recent studies have shown the efficiency of MgO NPs in the action against biofilms of *E. coli* (250 µg/mL), *K. pneumoniae* (125 µg/mL), and *S. aureus* (500 µg/mL). Bacterial adhesion to the plastic surface decreased markedly after 12 h incubation of *E. coli*, *S. aureus,* and *K. pneumonia* with MgO, thus preventing the biofilm formation. The effect of MgO on mature biofilms was also detected. Biofilm biomass was significantly reduced when treating biofilms with a subinhibitor concentration of 0.5 MIC. [83]. In another recent study, it was reported that 10 µg/mL significantly complicates the formation of *S. aureus* biofilms [115]. MgO NPs had a strong inhibitory effect on the formation of biofilms *E. coli* and *S. aureus* at a size of 8 nm [85]. MgO NPs reduced the biofilm growth of *R. solanacearu*, and the biofilm formation gradually decreased with the bulk MgO treatments. The 200 and 250 µg/mL treatments of MgO NPs exhibited high inhibitory effects on the *R. solanacearum* biofilm formation. The biofilm formation was reduced by 61% and 71% after 24 h and by 67% and 72% after 72 h, respectively [84]

Thus, MgO NPs have significant antibiofilm properties, but significant effects are achieved at sufficiently high particle concentrations (above 125 µg/mL).

### 3.4. TiO_2_

TiO_2_ NPs are effective against bacteria, viruses, and even to purify specific odor molecules in the range of 20 µg/mL to 1400 µg/mL [116,117,118,119,120]. TiO_2_ NPs show antibacterial properties against Gram-positive and Gram-negative, the latter being more sensitive [121,122]. It could be related to the fact that Gram-positive bacteria have a thick layer of peptidoglycan that facilitates the absorption of reactive radicals, thereby preventing cell damage from radical attack [123]. In addition, it shows that TiO_2_ has a potential against bacteria through the reception of an electron from intracellular coenzyme A (CoA) after photocatalysis of TiO_2_, followed by the formation of dimer CoA and subsequent inhibition of respiration [124].

TiO_2_ NPs can reduce the adhesion of bacteria and inhibit biofilms. Exposure to titanium oxide leads to the destruction of bacteria inside the biofilm, primarily due to the generation of ROS and lipid oxidation on the cell wall membrane [92,120]. It has been shown that TiO_2_ NPs are effective against biofilms of MRSA [92] and *S. mitis* [125]. TiO_2_ NPs could control the growth and biofilm formation of *S. mitis* ATCC 6249 and Ora-20, and it can be used as a means for oral hygiene. TiO_2_ NPs have a reduced impact on *Pseudomonas aeruginosa* biofilms at a 31.25 µg/mL concentration and disrupt previously established biofilms in the microtiter plate [125]. In the presence of TiO_2_ NPs, biofilm formation of *E. coli* and *B. subtilis* was reduced by 40–50% [122]. However, NPs TiO_2_ did not show significant bactericidal properties against certain types of drug-resistant bacteria (ex, *Cupriavidus metallidurans* CH34), which have a remarkable ability to withstand ROS membrane damage through overexpression of protective components and membrane repair elements [126].

### 3.5. Fe_3_O_4_

Fe_3_O_4_ NPs has slight antibacterial properties, and their effective concentrations reach 10–20 mg/mL [38,42,127,128]. Fe_3_O_4_ NPs against biofilms showed mostly insignificant effects. To obtain significant antibiofilm effects, Fe_3_O_4_ NPs have to be used in high concentrations. In this case, the particles are able to destroy the cells inside the biofilm. It has been shown that iron-oxide NPs were able to reduce biofilm growth by *S. aureus*, *E. coli*, *P. aeruginosa* [88], *S. epidermidis* [38], and *Enterococcus hirae* [89].

In addition to the passive electrostatic effect on biofilm, NPs effectively penetrate deep into the biofilms in the presence of a magnetic field [129,130]. In this case, NPs can have a mechanical effect on the biofilm due to the destruction of the matrix structure and its whole architecture [87,131,132]. Due to these properties, the particles are mainly used as a carrier of biocides in biofilms. In particular, the NPs enhance the action of various antibiotics on biofilm. The effectiveness of conjugates with penicillin, streptomycin, erythromycin, kanamycin, cefotaxime against *S. aureus* biofilm [133,134] and amphotericin B, nystatin against *Candida* spp. biofilm has been shown [135]. In recent years, many biocide-conjugated particles have been developed against biofilms. In all cases, magnetite serves as an efficient matrix for delivering biocides inside the biofilms and has a synergetic effect due to its unique properties.

Thus, Fe_3_O_4_ NPs have a less significant antibacterial property but have a significant antibiofilm potential due to the delivery of different antimicrobial drugs into the biofilm via a magnetic field [87,129,130,131,132,133,134,135,136].

### 3.6. Al_2_O_3_

Al_2_O_3_ NPs are effective against bacteria only when in high concentrations (reach 10–20 mg/mL) [137,138]. Al_2_O_3_ NPs weakly inhibit *E. coli* at high concentrations up to 1 mg/mL [137,138]. In the study of Al_2_O_3_ NPs’ effect on *P. putida* and *A. hydrophila* in biofilms and planktonic forms, it has been shown that NPs are toxic to bacteria, but plankton cells are more susceptible to Al_2_O_3_ NPs than biofilms [61]. The minimal inhibitory concentration of Al_2_O_3_ NPs against the biofilm of *P. aeruginosa* was found to be 1.6–3.2 mg/mL. Treatment at a 2 mg/mL concentration resulted in complete growth inhibition of extended-spectrum b-lactamases and metallo-b-lactamases clinical isolates of *P. aeruginosa* [139]. Therefore, antimicrobial and antibiofilm properties of Al_2_O_3_ are less than those of other oxides. Thus, it can only be effective in nanocomposites and conjugates with biocides.

It is clear from the above that metal oxide NPs can be promising materials against biofilms. Different particles exhibit antibiofilm properties to varying degrees, which depends directly on their antibacterial properties. These properties are determined mainly by the synthesis method, size, and shape of the particles (Table 1). Particles, such as Fe_3_O_4_ and Al_2_O_3_, have weak antibiofilm properties, but they can be much more effective in nanocomposites.

## 4. Metal Oxide Nanocomposites against Plankton Cells and Biofilms

It can be seen from the previous section that the antibiofilm properties of metal oxide NPs are demonstrated to varying degrees. Nanocomposites of mixed metal oxides are actively studied and developed to improve antimicrobial properties and reduce adverse cytotoxic effects and reactions of the immune system to monoxide NPs.

Nanocomposite CuO doped with Zn showed an increase in antibacterial activity of 10 times against *E. coli* and *S. aureus* bacteria compared to pure ZnO or CuO [140]. Moreover, CuO doped with TiO_2_ has a more significant antibacterial effect than pure TiO_2_. Li-doped MgO NPs are more efficient than pure MgO, whereas Zn and Ti-doped MgO exhibit lower antibacterial activity than MgO [22]. TiO_2_-ZnO-MgO mixed oxides nanomaterials have a strong antibacterial effect against Gram-negative and Gram-positive bacteria [141].

The combination of three metal oxides in the CuZnFe oxide NPs may also enhance the therapeutic abilities of the NPs against a wide range of microbial infections. Antibacterial activities of CuZnFe oxide NPs were tested against Gram-negative *E. coli* and Gram-positive *E. faecalis*. CuZnFe oxide NPs affected bacterial species by reducing their viability and their ability to synthesize biofilm. CuZnFe oxide NPs have more detrimental effects on *E. coli* than individual CuO and ZnO NPs. CuZnFe oxide NPs were also found to be more bactericidal than ZnO NPs against *E. faecalis*, but 7% lower than CuO NPs [94]. It was observed that this oxide could slowly release metal ions, which can penetrate through the membranes and disrupt cellular processes from within the cell [142]. CuZnFe oxide NPs affected biofilm formation to a lesser degree than those of individual ZnO and CuO NPs [94].

The pro-oxidative and pro-inflammatory effects of highly toxic ZnO NP can be significantly reduced by iron doping [143,144]. A similar effect can also be achieved by adding magnesium to the nanocomposite: mixed ZnMgO NPs, are safe for mammalian cells with the non-toxic MgO monoxide NPs [20]. ZnO NPs doped with Mn and Fe ions exhibit even higher antibacterial activity on a wide range of bacterial species, including *S. aureus*, *E. coli*, *K. pneumoniae*, *S. typhi*, *P. aeruginosa, B. subtilis*, and *Proteus mirabilis* as compared to the ZnO monoxide [21,145]. In the study of Fe_3_O_4_-ZnO nanocomposite, it has shown more significant antibacterial activity on *E. coli* than *S. aureus* and *B. subtilis* [145].

The inclusion of Ag and Au in composites also significantly enhances their antimicrobial properties. Ag-ZnO nanocomposites showed a high antibacterial effect against antibiotic-resistant *E. coli* and *S. aureus* [146]. High antibacterial activity against *E. coli* and *S. aureus* was shown by Ag-SiO_2_ nanocomposite. This composite is perfect for the treatment and infectious control of superficial wounds [96]. Moreover, the deposition of Au particles on the surface of ZnO NPs, even at a low molar ratio of ZnO/Au (0.2%), significantly improves the photocatalytic antibacterial activity of ZnO [147]. Furthermore, Ag-TiO_2_ nanocomposite demonstrates antibacterial activity against *S. aureus* biofilms [93,148,149]. It was shown that the inclusion of a 2% composite significantly reduced the formation of biofilms on the surface of the composite resin [150]. Ag/Fe_3_O_4_ NPs significantly improve antibacterial properties against *E. coli* [151]. CeO_2_-CdO nanocomposites also exhibit broad-spectrum antimicrobial activity against Gram-positive (*S. aureus* MTCC96 and *C. pyogenes* MTCC 1926) and Gram-negative (*P. aeruginosa* и *K. pneumoniae*) [95].

Additionally, the introduction of nanocomposites based on oxides induced by external physical factors can add additional functions and significantly increase antimicrobial action effectiveness. For example, compared to the monoxide TiO_2_ NPs, which exhibit photocatalytic activity in the UV spectrum, the doped form can significantly expand the active spectrum to the visible light region [152,153]. An example of a highly effective nanocomposite can be ZnOAu, which possesses photoactivity due to zinc, and thermal sensitivity due to gold [97].

Some nanocomposites have been studied in vivo. CuO ligated Zn shows a successful inhibition of biofilm formation in in vitro and in vivo experiments on rabbits. At the same time, this composite is biocompatible [81].

As shown in the above, the antibacterial activity of initially low-efficiency oxides (such as Fe_2_O_3_ NPs) in mixed oxides can be significantly increased. It becomes evident that oxide particles, and especially composites based on them, can become promising materials. Reducing their cytotoxicity and immunogenicity will also allow their introduction into biomedicine. Ultimately, the widespread application of nanomaterials also needs to be determined by their safety to the environment.

## 5. Potential Adverse Effects of the Broad Implementation of Metal Oxide NPs

Along with their numerous remarkable applications, NPs also have potential limitations. In particular, a large surface area and high reactivity can be considered as one of the NPs advantages, but at the same time cause side effects. Moreover, the non-specificity of the antimicrobial action on pathogenic and symbiotic microorganisms exhibited by NPs can be seen as an advantage or disadvantage [154]. Chemically synthesized NPs have toxicity problems since dangerous compounds are used in their synthesis. These toxic compounds remain within NP in trace amounts and cause undesirable effects [155]. A successful approach to solving these problems is developing methods to synthesize environmentally friendly and less toxic NPs—the so-called “green synthesis” [156].

The most important aspect of the safe use of nanostructures is their mutagenicity. Even though this issue remains the least studied, there are already some literature reports that raise concerns. As mentioned earlier, the NPs and ROS that they generate can themselves influence the DNA of bacteria. If the concentration of NPs is not sufficient to eradicate the biofilm, but enough to trigger mutations, which can lead to the appearance of “super mutants”.

The study of NPs genotoxicity using tungsten oxide as an example has shown that direct interaction NPs with DNA can lead to damage by single-strand breaks. After the mix with NPs DNA was introduced into bacterial cells, the cells mostly died, and the surviving cells were almost all mutants. The results provide clear evidence that one of the mechanisms involved in nanomaterials toxicity directly damages DNA, which can then cause biological cell death and mutation [157].

The potential mutagenicity of certain oxides (Al_2_O_3_, Co_3_O_4_, CuO, TiO_2_, and ZnO) was investigated using reverse mutation (Eames analysis). The results showed that the mutagenicity was negative for four nanoparticles (Al_2_O_3_, Co_3_O_4_, TiO_2_, and ZnO) to 1000 µg/plate for all three strains tested (*S. typhimurium* TA97a, TA100, and *E. coli* WP2 trpuvrA) without metabolic activation of S9. Using the pre-incubation procedure and high activation of S9 (9%), TiO_2_ and ZnO induced marginal mutagenesis for the *E. coli* strain WP2. The CuO exhibited a low mutagenic potential in *S. typhimurium* TA97a and TA100 at specific concentrations [158].

In addition to direct DNA damage under the influence of NPs, there are also data on increasing the efficiency of horizontal transfer of genetic material when exposed to NPs. It has been shown that Al_2_O_3_, AlOOH, TiO_2_, SiO_2_, and Fe_2_O_3_ NPs can contribute to the conjugating transfer of plasmid (up to 20–100 times) [159,160,161]. It is important to note that the transfer increased inside the species or genus of bacteria. The most significant effect was produced by Al_2_O_3_ NPs, which led not only to an increase in the number of conjugating cells but also to one bacterium being conjugated to several other bacteria [159].

Such manifestations of NPs mutagenicity in the biofilms of bacteria are especially important. Genetic components from lysed bacterial cells, such as plasmids, are stored inside the EPM, increasing the gene transfer frequency between bacterial cells [162]. These plasmids can contain genes useful for bacteria, such as genes for antibiotic resistance [163]. Thus, it can be assumed that metal oxide NPs can contribute to the formation of antibiotic-resistant bacteria. Further research on the issues described above is essential for the safe use of NPs.

An essential aspect of the widespread introduction of nanostructures is the ecological one. Currently, metal oxide NPs are already widely used in various fields. The presence of nanomaterials in the environment is not new. Nevertheless, the current growth in the production of anthropogenic nanomaterials will increase their level in the environment. The release of nanoparticles into the biosphere will occur from point sources (for example, production sites, landfills, treatment plants) and secondary sources (for example, release into the environment when using and consuming materials containing nanomaterials). For example, a global estimate for nanomaterial production goes up to 5000 tons/year for TiO_2_ NPs [164].

There is a gradual increase in the concentration of these NPs in wastewater as contaminants. This can threaten ecological communities in treatment plants [165]. Recent studies have shown that significant accumulation of NPs is observed in biofilms of the river and marine sediments [166,167]. A high concentration of 20 nm TiO_2_ is found in river biocenoses [166]. NP can pose a danger to the environment, causing the disappearance of some biocidal sensitive bacterial strains and expanding a set of insensitive strains to various influences [126].

The properties of NPs described in this section show that currently, available research data are insufficient to assess the safety of NPs use. More studies need to be carried out before these nanomaterials can enter the broad market.

## 6. Conclusions

In addition to the biofilms spread, which are resistant to antibiotics, the spread of biofilms produced by antibiotic-resistant bacteria grows. Using metal oxide NPs and their nanocomposites is one of the few methods that can soon enter into broad practice.

Among the NPs discussed in this review, CuO and ZnO NPs showed the most significant antibiofilm properties, which initially had high antibacterial characteristics. These NPs have proven effective against biofilms of a wide range of bacterial species. Fe_3_O_4_, TiO_2_, and MgO NPs have a lower ability to eradicate biofilms. However, magnetite can be used with a magnetic field to deliver biocides and has shown high biofilm destruction efficiency. Al_2_O_3_ NPs have the weakest properties against biofilms, and these particles can only be effective in high concentrations. However, the introduction of nanocomposites consisting of several oxides can significantly enhance the action of NPs against biofilms. Several of the composites described in this review have excellent antibacterial and antibiofilm properties.

However, the mechanisms of NPs action on biofilms are still weakly studied and controversial. A more thorough study of the mechanisms of NPs’ action on plankton cells and biofilms may allow more targeted use and increase their effectiveness. Another issue is the potential particle mutagenicity, resulting from which bacteria can acquire new dangerous properties by analogy with the development and spread of antibiotic-resistant strains. Under conditions of mixed cultures of bacteria, especially in biofilms, NPs can, hypothetically, accelerate bacterial evolution. Nevertheless, this aspect of the NP influence on prokaryotes is the weakest studied.

It can be concluded that, despite extensive research on NPs worldwide, many issues require more in-depth studies. Without them, nanostructured drugs introduction to therapy can have unpredictable consequences for both the evolution of bacteria and the human body. The use of NPs on the industrial level can also provoke severe environmental consequences.

Thus, the current review formulates specific issues that have to be addressed to push the research forward. Since it is essential to develop new approaches for combating biofilms of bacteria in various fields, new information on the effectiveness of the use of NPs against biofilms, their mechanisms of influence on cells, mutagenicity, and genotoxicity will be crucial.

## Figures and Tables

**Table 1:** Physical and antibiofilm properties of metal oxide nanoparticles (NPs) and nanocomposites.

NP	Synthesis	Physical Properties	Antibiofilm Properties Against Species:	Reference
Al_2_O_3_	Purchased at the Sigma–Aldrich, no information about the synthesis method	50 nm	*P.putida*, *Aeromonashydrophila*	[61]
CuO	Sonochemical method	40 nm	*Candida* sp.	[62]
CuO	Green synthesis	50 nm	*E. coli*, *P. aeruginosa*, *K. pneumonia*, *P. vulgaris*	[63]
CuO	Green synthesis	No information	*E. coli* ATCC 25922, *S. aureus* ATCC 45500	[64]
CuO	Chemical reduction method	20 ± 1.24 nm	*E. coli*, *P. aeruginosa*, *B. subtilis*, *S. aureus*	[65]
ZnO	Wet chemical method	~15 nm	*S. pneumoniae* MTCC 2672	[11]
ZnO	Purchased at the Sigma–Aldrich, no information about the synthesis method	<50 nm	*P. aeruginosa PAO1*	[66]
ZnO	Green synthesis	10–50 nm, triangles, hexagons, rods, and rectangles	*B. licheniformis*, *B. pumilus*, *E. coli*, *P. vulgaris*	[67]
ZnO	Wet chemical method	7–10 nm	Classical (O395) and ElTor (N16961) *V. cholerae*	[68]
ZnO	No information	65 nm	*P. aerugonosa*	[69]
ZnO	Green synthesis	8–18 nm, spherical, oval, hexagonal	*E. coli*, *P. aeruginosa* (ESBL), MRSA, MSSA	[70]
ZnO	Green synthesis	20–50 nm, spherical and hexagonal	MRSA	[71]
ZnO	Purchased at the Sigma–Aldrich, no information about the synthesis method	50–500 nm, spherical	*M. smegmatis*, *M. bovis BCG*, *E. coli*, *P. aeruginosa*, *S. aureus*, *MRSA*	[72]
ZnO	Green synthesis	90–100 nm, triangles, hexagons, rods, and rectangle	*V. cholerae*, *E. coli* (ETEC)	[73]
ZnO	Purchased at the Sigma–Aldrich, no information about the synthesis method	~100 nm	MRSA	[74]
ZnO	No information	20 nm, plates, spheres, pyramids	*E. coli* UTI89 and MG1655, *K. pneumoniae* LM21, MRSA SH1000, *S. epidermidis* RP62A	[75]
ZnO	No information	20 nm	*E. coli*, *S. aureus*, *S. sobrinus* ATCC 27352, *Enterobacter* sp., *Marinobacter* sp.	[76]
ZnO	Green synthesis	26 nm	*C. violaceum* 12472, *C. violaceum* CVO26, *E. coli*, *L. monocytogenes*, *P. aeruginosa* PAO1	[77]
ZnO	Microwave radiation	No information	*C. violaceum* ATCC 12472, *E. coli* ATCC 25922, *P. aeruginosa* PAO1, *K. pneumoniae* ATCC 700603, *S. marcescens* ATCC 13880	[78]
ZnO	Sol-gel method	No information	*R. dentocariosa*, *R. mucilaginosa*	[79]
ZnO	Purchased at US Research Nanomaterials Co, no information about the synthesis method	10–30 nm	Uropathogenic *E. coli*	[80]
Zn-doped CuO	Sonochemical method	No information	*E. coli* ATCC 25922, *S. aureus* ATCC 29213, *P. mirabilis*	[81]
CeO_2_	Sonochemical method	~100 nm	*Periphytic biofilm*	[82]
MgO	Chemical synthesis	No information	*K. pneumoniae* KT273996, *E. coli* KT273995	[83]
MgO	Purchased at the Sigma–Aldrich, no information about the synthesis method	No information	*R. solanacearum*	[84]
MgO	Microwave radiation	No information	*E. coli,* *S. aureus*	[85]
NiO	Green synthesis, Eucalyptus globulus leaf extract	19 nm	ESβL (+) *E. coli*, *P. aeruginosa*, methicillin-sensitive and resistant *S. aureus*	[86]
Fe_3_O_4_	No information	No information	*B. subtilis* (ATCC 6633)	[87]
Fe_3_O_4_	Wet chemical method	10 nm	*S. aureus*, *P. aeruginosa*, *E. coli*	[88]
Fe_3_O_4_	Co-precipitation method	10.64 ± 4.73 nm	*E. coli* BW 25113, *E. hirae* ATCC 9790	[89]
hematite (α-Fe_2_O_3_)	No information	2 to 540 nm	*P. aeruginosa* (PA01)	[90]
TiO_2_	Sol–gel method	No information	*B. subtilis* strain 168	[91]
TiO_2_	No information	<100 nm	MRSA biofilm	[74]
WO_2_	Acid precipitation routes	No information	*B. subtilis* strain 168	[91]
TiO_2_	Green synthesis, by bacteria	10 to 30 nm, spherical	*B. subtilis* (FJ460362)	[92]
TiO_2_	Purchased at the Sigma–Aldrich, no information about the method of obtaining	<50 nm	MRSA	[74]
Ag–TiO_2_	Chemical reduction	31.3 ± 0.5 or 23.4 ± 0.4 nm, spherical or quasi-spherical	*B. subtilis*, *S. aureus*, *E. coli*, *K. pneumoniae*, *P. aeruginosa*, *Candida albicans*	[93]
ZnMgO	Burning corresponding metal ribbons	10 nm	*E. coli,* *B. subtilis*	[20]
CuO/TiO_2_	Sol–gel method	6 nm	*E. coli* ATCC 25922	[22]
MgO dopped Li	Sol–gel method	23 nm	*E. coli* ATCC 25922	[22]
CuZnFe	From solutions of metals	42 nm	*E. coli*, *E. faecalis*	[94]
CeO_2_-CdO	Green synthesis	10 nm	*P. aeruginosa* MTCC73	[95]
Ag-SiO_2_	Stöber method	670 nm	MRSA, *E*. *coli*	[96]
ZnOAu	Green synthesis	30 nm	*S. aureus*, *E. coli*	[97]
